# Training Load Distribution Across Weekly Microcycles According to the Match Schedule During the Regular Season in a Professional Rink Hockey Team

**DOI:** 10.3390/jfmk11010016

**Published:** 2025-12-29

**Authors:** Matteo Fortunati, Patrik Drid, Renato Baptista, Massimiliano Febbi, Venere Quintiero, Giuseppe D’Antona, Oscar Crisafulli

**Affiliations:** 1 Centro di Ricerca Interdipartimentale Attività Motorie e Sportive (CRIAMS)-Sport Medicine Centre Voghera, University of Pavia, 27058 Voghera, Italy; matteo.fortunati01@gmail.com (M.F.); venere.quintiero01@universitadipavia.it (V.Q.); oscar.crisafulli@unipv.it (O.C.); 2 Department of Industrial Engineering, University of Tor Vergata, 00133 Rome, Italy; massimilianofebbi@gmail.com; 3 Faculty of Sport and Physical Education, University of Novi Sad, 21000 Novi Sad, Serbia; patrikdrid@gmail.com; 4 Department of Research and Development, Lunex International University of Health, Exercise and Sports, Avenue du Parc Des Sports, 50, 4671 Differdange, Luxembourg; 5 Luxembourg Health & Sport Sciences Research Institute ASBL, Avenue du Parc des Sports, 50, 4671 Differdange, Luxembourg; 6 Laboratory for Rehabilitation, Medicine and Sport (LARM), 00133 Roma, Italy; 7 Department of Public Health, Experimental and Forensic Medicine, University of Pavia, 27100 Pavia, Italy

**Keywords:** team sport, training load, periodisation, conditioning, training prescription, heart rate

## Abstract

**Background**. This study aimed to quantify differences in the internal training load (ITL) of an elite rink hockey (RH) team across days within and between three types of microcycles: pre-season, in-season regular, and in-season congested, to provide insights to optimise microcycle scheduling. **Methods**. One international-level male RH team comprising seven outfielders (29.6 ± 4.7 years; height, 178.9 ± 2.3 cm; body mass, 77.8 ± 5.7 kg) and one goalkeeper (32 years; height, 180.4 cm; body mass, 83.6 kg) was monitored for 21 microcycles. The ITL was assessed using the session rate of perceived exertion (sRPE) and quantified as time based on a triphasic classification commonly utilised in team sports: low-intensity training (LIT, <80% heart rate maximum (HRmax)), medium-intensity training (MIT, 80–90% HRmax), and high-intensity training (HIT, >90% HRmax). Generalized estimating equations were used to examine differences across within-microcycle training days and between seasonal phases, with linear mixed models applied as sensitivity analyses. **Results**. Across all phases, significant day-to-day variations in ITL were observed within microcycles (all *p* < 0.001), with both subjective (sRPE) and objective (LIT–HIT) ITLs progressively decreasing as match days (MDs) approached, showing moderate-to-large population-averaged effects with 95% confidence intervals consistently not crossing zero. The pre-season exhibited the highest overall ITL (*p* < 0.001), characterised by a substantially greater sRPE and increased time spent across all intensity zones, with the largest magnitudes observed for LIT and MIT compared with the in-season phases. **Conclusions**. Findings suggest that an international-level RH team progressively reduced the ITL as MDs approached with the highest loads scheduled earlier within microcycles. Moreover, the pre-season had the highest ITLs. This ITL distribution may provide useful guidance for RH coaches and support staff in optimising microcycle planning.

## 1. Introduction

Rink hockey (RH) is a sport played by a team composed of four outfield players and one goalkeeper within a rectangular playing area measuring 40 m in length and 20 m in width. Outfield players perform frequent high-intensity intermittent actions, along with collisions, technical actions, and tactical transitions to score a goal [1,2,3,4]. In contrast, a goalkeeper’s role is to defend the goal, preventing the opposing team from scoring, and his actions typically involve short, explosive movements and dives [5]. Matches are played over two 25 min halves of effective time, with the clock stopped during referee interventions, increasing the duration of the competitions up to ~84 min [3]. A complete regular season could comprise a maximum of 55 competitive matches: 26 from the regular season, 13 from play-offs, 4 national cup matches, and 12 international matches (i.e., Euroleague). Therefore, a top team commonly faces a congested calendar, namely with up to two matches per week (i.e., microcycle). As already evidenced in professional soccer [6] and basketball [7], recognition of the workload challenges posed by such scheduling can inform coaching staff in managing training periodisation and programming, for example, by optimising training schedules and balancing low- and high-intensity sessions within weekly microcycles to maximise performance and recovery [7,8,9,10,11,12,13]. This scenario underscores the importance of carefully monitoring and managing training load (TL) to minimise fatigue and prevent underperformance [14,15].

The TL represents the overall stress imposed on an athlete by a given training session or competition, and it can be divided into external and internal components [16]. While external load refers to the measurable work performed, the internal training load (ITL) reflects individual physiological and psychological responses to that work [16]. The ITL can be quantified using objective physiological measures such as heart rate (HR) response, including time spent in predefined intensity zones, which provides information on cardiovascular stress [3]. In parallel, the subjective ITL captures the athlete’s perceived effort and overall experience of the session, commonly measured using the session rating of perceived exertion (sRPE) [17]. The sRPE method involves the athlete rating the intensity of the entire session on a standardised scale, typically Borg’s CR-10 [18], and multiplying this value by the session duration to generate a single index of the ITL [17]. Together, HR-derived metrics and sRPE provide complementary information: the former quantifies physiological stress objectively, while the latter integrates the athlete’s perception of effort, fatigue, and psychological strain [19].

From a physiological and molecular standpoint, TL fluctuations modulate several mechanisms that underpin performance in intermittent high-intensity sports. Repeated high-intensity efforts stimulate mitochondrial biogenesis through pathways such as AMPK–PGC-1α, enhancing oxidative capacity and metabolic efficiency [20], while moderate- and low-intensity work contribute to improved aerobic function and autonomic balance [21]. Conversely, insufficient recovery between high-load sessions may disrupt homeostasis, leading to increased sympathetic activation, impaired hormonal regulation (e.g., altered cortisol–testosterone dynamics), and reduced muscle repair capacity driven by inflammatory and neuromuscular fatigue processes [22,23]. Therefore, the weekly alternation of high- and low-intensity TL sessions plays a crucial role in promoting favourable adaptations while avoiding maladaptive responses such as excessive fatigue or overreaching [24]. In elite seasonal sports, microcycles are therefore structured to progressively manipulate the load across the week, commonly following a pattern of load accumulation, a peak stimulus, and subsequent tapering, to support both adaptation and readiness for competition [24].

Moreover, across the competitive year, training cycles are structured to reflect both the seasonal phase and the athletes’ physiological needs, with the overarching goal of maximising adaptation while preventing accumulated fatigue and overtraining. During the pre-season, higher training volumes and elevated ITL are typically prescribed to stimulate broad physical conditioning, enhance metabolic and neuromuscular capacities, and consolidate team tactical principles in the absence of competitive constraints [25,26]. As the season begins, training content transitions toward a more regulated weekly microcycle in which the load is progressively tapered as match day approaches, allowing athletes to maintain performance readiness. In regular in-season microcycles, coaches commonly schedule the highest loads early in the week, followed by gradual reductions to facilitate recovery and optimise match preparation [27,28]. During congested weeks, when two competitive matches occur, the training load is further adjusted by prioritising recovery, reducing high-intensity stimuli, and limiting cumulative stress [29]. These cyclical adjustments are essential for balancing stimulus and recovery, mitigating the risk of overreaching, and ensuring that athletes sustain high performance levels throughout the season.

Despite the growing emphasis on TL management in intermittent team sports [8,30,31], the current state of the art shows a lack of evidence specifically addressing how elite RH teams distribute ITL across different microcycles and how training and match demands vary day by day [32]. This lack of evidence limits not only the understanding of RH-specific physiological demands but also the capacity of practitioners to make informed day-to-day periodisation and prescription decisions. In particular, without data describing how ITL and sRPE fluctuate across the days of a microcycle, coaches may find it difficult to appropriately structure session contents, sequence training intensities, and balance load accumulation and recovery. To date, the only investigation on this topic indicates that the day preceding match day (MD-1) is typically characterised by a deliberately reduced TL [32]. Consequently, RH-specific ITL patterns are essential to guide the design of technical–tactical sessions, conditioning drills, and tapering strategies within weekly planning. Therefore, the present study aimed to investigate both the objective and subjective ITL in an international-level RH team competing in the National Cup, National Championship, and the European League during the 2022–2023 season. The primary objective was to quantify differences in ITL across days within three types of microcycles: pre-season, in-season regular (one match per week), and in-season congested (two matches per week). A secondary objective was to compare weekly ITL between these macrocycle types specifically for outfielders only. The scope of this study was to report and describe how an international-level RH team distributed and managed ITL throughout the season, providing practical insights to support coaches and performance staff in optimising training prescription.

## 2. Materials and Methods

### 2.1. Study Design

**Figure 1:**
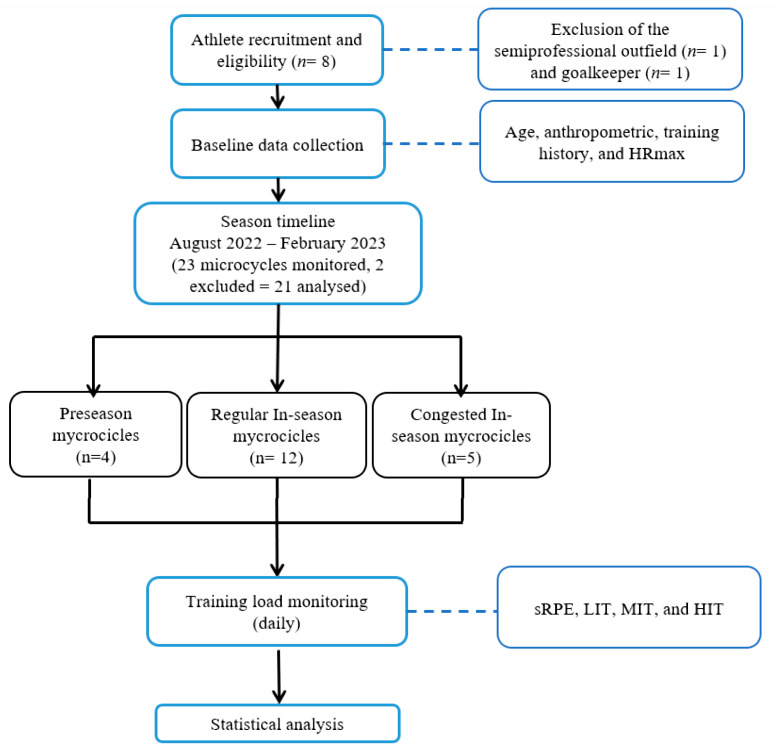
Flow chart of the study. *n*: number, sRPE: session rating of perceived exertion, LIT: low-intensity training, MIT: moderate-intensity training, HIT: high-intensity training.

The present study employs a non-experimental, observational, descriptive, and longitudinal design [33] drawn from a convenience sample of a single elite team based in northern Italy. Consequently, no sessions or games were modified in any way. All training sessions were planned by the head coach in collaboration with the performance and medical staff. Figure 1 presents the flowchart of the study, outlining the progression from athlete recruitment through training load monitoring to data processing and statistical analyses.

### 2.2. Subjects

Seven senior RH international outfielders (age, 29.6 ± 4.7 years; height, 178.9 ± 2.3 cm; body mass, 77.8 ± 5.7 kg) and one goalkeeper (age, 32 years; height, 180.4 cm; body mass, 83.6 kg) were monitored during the study period. Three athletes, including the goalkeeper, were members of their respective national teams. All the aforementioned athletes followed the same training schedule. The inclusion criteria were that athletes were members of the main team of the squad under investigation and were free from injuries that could have compromised training and/or match participation. The exclusion criteria were athletes who did not attend all team trainings and matches, as well as technical failures of the monitoring system. For this reason, the semi-professional goalkeeper and one outfield player were excluded from the study.

A registered nutritionist and sports psychologist followed all players throughout the study period. All athletes played in a team competing in the major National RH league and the Euroleague. Since the 2016–2017 season, the team has won three National Championships and consistently qualified for the playoffs in the following years; therefore, it can be considered one of the best representatives of the professional RH panorama. During the period monitored, no cases of underperformance due to overtraining [34], burnout [35], overuse injuries [36], and relative energy deficiency [37] were identified, and there were no personal absences from sports duty during this time.

The study was approved by the Ethics Committee of the Faculty of Sport and Physical Education, University of Novi Sad, Serbia (Ref. n°: 47-06-02/2021-2) and was conducted in accordance with the Declaration of Helsinki. All athletes provided written consent for analysis and publication of their data.

### 2.3. Subdivision of the Season

The monitored season period began in late August 2022 and ended in February 2023. The analysed period was selected to include a number of microcycles consistent with previous investigations on TL in RH [32]. During this period, the team competed in the National Championship, the National Cup, and the Euroleague group stage. Four weeks coincided with the 2022 World Championship (7–13 November), during which national team members trained and competed with their respective teams, while the remaining squad participated with the U19 team; these weeks were therefore excluded from daily monitoring. In total, 23 microcycles were initially recorded, of which 2 were excluded as they fell during the Christmas–Epiphany break and could not be grouped. Consequently, 21 microcycles were analysed and classified as follows: 4 pre-season microcycles, 12 in-season regular microcycles with one match per week (Saturday) comprising National and Euroleague Championships, and 5 in-season congested microcycles with two matches per week (Wednesday and Saturday).

The pre-season microcycles consisted of five days of double training RH practice sessions (Monday–Friday) and one day of single training sessions (Saturday). Notably, considering the absence of match days (MDs), the weekly schedule is reported with the days of the week. The in-season regular microcycles consisted of five consecutive days of training classified according to the day on which they were conducted with respect to MD [12] as MD+2, +3, -3, -2, and -1. A double session was performed on both MD+2 and MD+3. The in-season congested microcycles comprised three days of training (MD+2/-2, MD-1, and MD+2/-1) and two matches (MD1 and MD2). Notably, after MD1, there was a full day off. A detailed description of the training days is reported in Table 1.

### 2.4. Anthropometry Assessment

Height was measured after a deep breath using a wall-mounted stadiometer (Seca, model 220, Hamburg, Germany) rounded to the nearest 0.1 cm, while bodyweight was measured standing on a professional scale (Seca, model 711, Hamburg, Germany) and rounded to the nearest 0.1 kg.

### 2.5. Training Load Quantification

#### 2.5.1. Subjective Internal Training Load

Subjective ITL was quantified by the sRPE. Approximately 30 min after training sessions and games in their resting room [17], each athlete individually reported how difficult they felt the RH practice or match on a Borg CR-10 scale [18]. All responses were uploaded to the team’s athlete monitoring database, where each sRPE value was multiplied by the session duration to generate an ITL value [17]. sRPE is a valid measure of ITL across a range of training modalities and team sports [38,39,40,41] and is commonly used in RH [32].

#### 2.5.2. Objective Internal Training Load

Objective ITL was quantified by monitoring HR during training sessions (RI practice) and matches. Before each activity, players wore an HR chest strap (Polar H10^®^, Kempele, Finland) connected to the free version of the Polar Team system (Polar^®^, Kempele, Finland), with data sampled at 5 Hz [3,42,43]. Maximal heart rate (HRmax) was individually determined from two sessions of the 20-metre multi-stage shuttle roller skate test [44], performed 96 h apart, during the first week of preseason. Specifically, athletes were required to skate, using their own roller skates, between two lines that were 20 m apart, starting at 8.5 km h^−1^ and increased their speed by 0.5 km h^−1^ each minute. Speed was dictated by an audio signal from a computer. All subjects were verbally encouraged to perform maximally during the test. The protocol consisted of skating from the start line to the parallel line, turning, and skating back to the start line in time with the signals emitted from the speaker computer. Subjects continued this pattern of shuttle skating until they could no longer skate (volitional exhaustion) or they failed to reach the line in time with the speaker signals on two successive occasions (disqualification) [44]. HRmax was defined as the peak heart rate recorded during the test. Subsequent measurements of HRmax during training sessions and matches did not differ from the values obtained in the test. For the monitoring procedure, regarding RH practice, HR recording began at the start of the warm-up and continued until the end of the session, excluding cool-down because it only entailed stretching. In matches, recording began with the effective start of each half and ended at its conclusion; intermissions were excluded, while time-outs and referee stoppages were included. No technical failures were observed during the monitored period.

The HR zones, which are by default as follows: Zone 5 (>90% HRmax), Zone 4 (80–90% HRmax), Zone 3 (70–80% HRmax), Zone 2 (60–70% HRmax), and Zone 1 (<60% HRmax), represent the time (minutes and seconds) an athlete spent maintaining a given HR relative to his measured HRmax [45]. For a triphasic classification, Zone 1, which was the sum of Zone 1- to -3 (<80% HRmax) was defined as low-intensity training (LIT), Zone 2, which comprised Zone 4 (80–90% HRmax), was defined as medium-intensity training (MIT), and Zone 3, which represented Zone 5 (>90% HRmax), was defined as high-intensity training (HIT). A triphasic repartition of the intensity using the HR zones has been commonly utilised in other investigations regarding team sports, e.g., soccer [46,47,48] and Australian football [49].

### 2.6. Statistical Analysis

Data are presented as estimated marginal means (EMMs) ± standard error (SE) and 95% confidence intervals (95% CI). Considering the same microcycle type, the in-season National Championship and Euroleague were merged. Generalized estimating equations (GEEs) were used as the primary analytical approach. For each variable (sRPE, LIT, MIT, and HIT), GEE models included season phase-specific weeks (pre-season, regular in-season, or congested in-season weeks) and within-microcycle training days as fixed factors, as well as their interaction. Player identity was specified as the clustering variable to account for repeated observations within individuals. An unstructured working correlation matrix was selected to model within-player correlations across repeated measurements flexibly. Statistical significance of model effects was assessed using Wald chi-square tests. Where significant main or interaction effects were observed, estimated marginal means (EMMs) were computed to aid interpretation and to describe the distribution of ITLs across microcycles and training days. To assess the robustness of the findings, linear mixed models (LMMs) were conducted as a sensitivity analysis for all ITL variables and season phases. LMMs were estimated using restricted maximum likelihood (REML), and degrees of freedom for fixed effects were calculated using the Satterthwaite approximation. Model results were reported as F-statistics with corresponding degrees of freedom and *p*-values. Model explanatory power was quantified using marginal R^2^ (variance explained by fixed effects) and conditional R^2^ (variance explained by both fixed and random effects). To evaluate whether statistically significant differences detected by the GEE models were also practically meaningful, the smallest worthwhile change (SWC) was calculated for each load variable (sRPE, LIT, MIT, and HIT). Consistent with established magnitude-based decision thresholds, the SWC was defined as 0.2× the typical within-athlete variability. To obtain this value, the dataset was first segmented by athlete, microcycles, and macrocycles. For each athlete, the within-week standard deviation of each variable was computed across the microcycle sessions, providing an estimate of the day-to-day variation that normally occurs under stable conditions. These weekly SD values were then averaged within each athlete, yielding that athlete’s typical variation across the macrocycles. Finally, the within-athlete SDs were averaged across all athletes to generate a single group-level estimate. This calculation was performed for each macrocycle, resulting in a macrocycle’s specific SWC. Of note, considering that the goalkeeper was just one individual, his ITLs are presented as purely descriptive, without an inferential purpose.

To evaluate the difference between macrocycles’ ITLs, weekly sRPE and HR-derived variables (LIT, MIT, and HIT) were analysed using GEE with an exchangeable working correlation structure, with robust (sandwich) standard errors. Additionally, LMMs as sensitivity analysis were conducted. Models were estimated using restricted maximum likelihood (REML), and degrees of freedom were approximated using the Satterthwaite method.

Agreement between HRmax obtained during the skating shuttle test and weekly HRmax was assessed using a linear mixed-effects model, including HRmax values recorded during two training days (Mon and Sat for preseason), one training day (MD+2) and MDs for the regular in-season, and both MDs in congested in-season. This approach was adopted to account for repeated measurements within subjects, as the assumption of independence required for the classical Bland–Altman method was violated. The 95% limits of agreement (LoA) were calculated using the total variance derived from the mixed-effects model:

$$SD_{total}=\sqrt{\sigma_{between}^{2}+\sigma_{within}^{2}}$$

$$LoA=\mu \pm 1.96\times SD_{total}$$

where $\sigma_{between}^{2}$ represents between-subject variance and $\sigma_{within}^{2}$ represents within-subject (residual) variance.

Internal validation and reproducibility of the sRPE protocol were examined using complementary correlational and reliability-based approaches. Firstly, an HR-derived ITL (modified TRIMP) was calculated as the weighted sum of time spent in the three HR zones, according to the following formula:

Modified TRIMP = (LIT × 1) + (MIT × 2) + (HIT × 3)

Then, to assess the internal validity of the sRPE protocol, the association between sRPE and the TRIMPmod was examined using Spearman’s rank correlation coefficient (ρ). Spearman’s rho was selected in preference to Pearson’s correlation because sRPE represents an ordinal perceptual measure, the data showed a longitudinal repeated-measures structure, and Spearman’s rho provides a robust, non-parametric estimate that is less sensitive to deviations from normality and outliers. The correlation analysis was first conducted across all available training sessions, pooling data from all players. Additionally, to examine whether the relationship between sRPE and TRIMPmod was consistent within individual players, the same Spearman correlation analysis was repeated at the subject level.

The reproducibility of sRPE responses was examined by estimating intraclass-type reliability (ICC) across repeated training sessions within all macrocycles, focusing on sessions characterised by similar contextual demands (e.g., Mon, Tue., etc.; MD, MD+2, etc.). Statistical significance was set at *p* < 0.05 for all analyses. The magnitude of correlation coefficients was interpreted according to commonly used thresholds in sports science research: <0.30: small, 0.30–0.49: moderate, 0.50–0.69: large, and ≥0.70: very large. Reliability coefficients were interpreted as follows: <0.50: poor, 0.50–0.74: moderate, 0.75–0.89: good, and ≥0.90: excellent. Analysis was performed with IBM SPSS Statistics v 31.0.1, 2025 (IBM Corp., Chicago, IL, USA) and Microsoft Excel^®^, v.2511 (Microsoft, Redmond, WA, USA).

## 3. Results

The individual athletes’ characteristics are reported in Table 2.

Overall, no athlete missed any training session or match during the monitored microcycles; therefore, a total of 121 unique observations were collected across the eight participants. Specifically, four observations were collected for each training day during the preseason (Mon to Sat), twelve observations were collected per training day during the regular in-season (MD+2, MD+3, MD-3, and MD-2), five observations were collected per training day during the congested in-season (MD+2/-2, MD+1/-1), and 17 and 22 observations were collected for MD-1 and MD, respectively, throughout the in-season phases.

### 3.1. Within-Microcycles’ Internal Training Load Comparisons

#### 3.1.1. Pre-Season Microcycles

**Figure 2:**
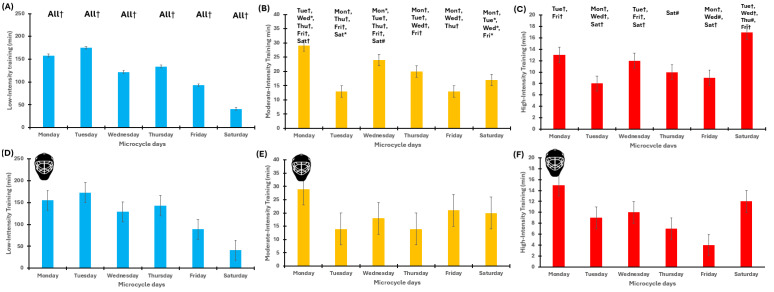
Within pre-season microcycles’ internal training load for outfielders and the goalkeeper. Panel (**A**–**C**): internal training load of outfielders during the pre-season microcycles. Panel (**A**) represents LIT, Panel (**B**) represents MIT, and Panel (**C**) represents HIT. Panel (**D**–**F**): internal training load of the goalkeeper during the in-season congested microcycles. Panel (**D**) represents LIT, Panel (**E**) represents MIT, and Panel (**F**) represents HIT. MD: match days. Colours: light blue, low-intensity training; orange, medium-intensity training; red, high-intensity training. The goalkeeper is indicated by the helmet. Symbols: * *p* < 0.05, # *p* < 0.01, † *p* < 0.001.

The GEE model revealed significant effects of microcycle (*p* < 0.001), training day (*p* < 0.001), and their interaction (*p* < 0.001) for all the ITL variables. Specifically, the highest values were observed on Monday and Wednesday, while the lowest occurred on Saturday and Friday. LIT varied significantly, with peaks on Tuesday and Monday and minima on Saturday. Regarding MIT, its peaks occurred on Monday and Wednesday, with minima on Friday. Finally, HIT was the highest on Saturday and Monday, with minima on Friday and Tuesday (Table 3). The sensitivity analysis conducted for all ITL variables using LMMs confirmed these findings. To evaluate practical significance, the SWC values for sRPE, LIT, MIT, and HIT were, respectively, 46 AU, 12 min, 2.25 min, and 1.3 min. Most of the comparisons of ITLs exceed SWC and thus have practical value. A detailed description of the results is provided in the Supplementary Materials and Supplementary Table S1. A graphical representation is presented in Figure 2.

#### 3.1.2. In-Season Regular Microcycles

**Figure 3:**
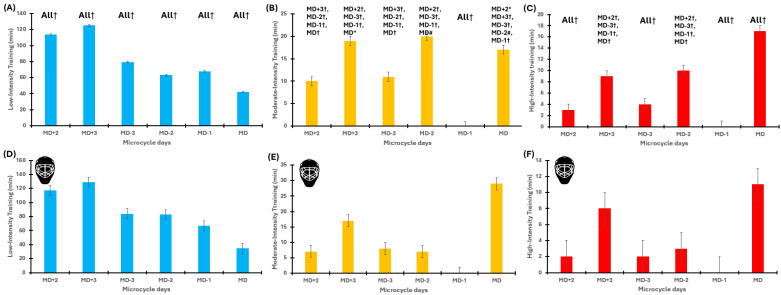
Within-regular in-season microcycles’ internal training load for outfielders and the goalkeeper. Panel (**A**–**C**): internal training load of outfielders during the in-season regular microcycles. Panel (**A**) represents LIT, Panel (**B**) represents MIT, and Panel (**C**) represents HIT. Panel (**D**–**F**): internal training load of the goalkeeper during the in-season congested microcycles. Panel (**D**) represents LIT, Panel (**E**) represents MIT, and Panel (**F**) represents HIT. MD: match days. Colours: light blue, low-intensity training; orange, medium-intensity training; red, high-intensity training. The goalkeeper is indicated by the helmet. Symbols: * *p* < 0.05, # *p* < 0.01, † *p* < 0.001.

The GEE model revealed significant effects of microcycle (*p* < 0.001), training day (*p* < 0.001), and their interaction (*p* < 0.001) for all the ITL variables. Specifically, sRPE was systematically lower on days MD−1 and MD−3 and substantially higher on MD+2 and MD+3. LIT was consistently higher on post-match days (MD+2 and MD+3) and lower on MD. MIT was higher on post-match days (MD+2 and MD+3) and had lower values on MD−1. Finally, HIT was predominantly accumulated on MDs and early post-match sessions (MD+2), with consistently lower values observed on the day preceding a competition (MD−1) (Table 4). The LMM fully confirmed the GEE results regarding all ITL variables. To evaluate practical significance, the SWC values for sRPE, LIT, MIT, and HIT were, respectively, 52 AU, 7.4 min, 2.1 min, and 1.4 min. Most of the comparisons exceed SWC and thus have practical value. A detailed description of the results is provided in the Supplementary Materials and Supplementary Table S2. A graphical representation is presented in Figure 3.

#### 3.1.3. In-Season Congested Microcycles

**Figure 4:**
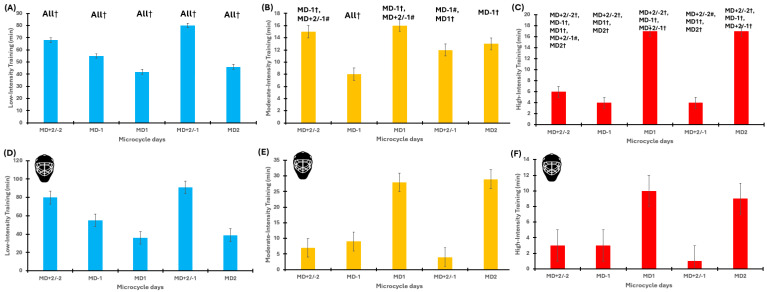
Within-congested in-season microcycles’ internal training load for outfielders and the goalkeeper. Panel (**A**–**C**): internal training load of outfielders during the in-season congested microcycles. Panel (**A**) represents LIT, Panel (**B**) represents MIT, and Panel (**C**) represents HIT. Panel (**D**–**F**): internal training load of the goalkeeper during the in-season congested microcycles. Panel (**D**) represents LIT, Panel (**E**) represents MIT, and Panel (**F**) represents HIT. MD: match days. Colours: light blue, low-intensity training; orange, medium-intensity training; red, high-intensity training. The goalkeeper is indicated by the helmet Symbols: # *p* < 0.01, † *p* < 0.001.

The GEE model revealed significant effects of microcycle (*p* < 0.001), training day (*p* < 0.001), and their interaction (*p* < 0.001) for all the ITL variables. Specifically, sRPE was generally the highest on MDs and for early post-match sessions, while markedly lower values were observed on the pre-MD. LIT values were highest on MD+2/−1 and MD+2/−2, while MDs were characterised by substantially lower accumulation (all *p* < 0.001). The highest MIT loads occurred on MD+2/−1 and MDs, whereas MD−1 and MD+2/−2 were associated with substantially lower expositions. The highest values of HIT were observed during both MDs compared with other training days (Table 5). The LMM fully confirmed the GEE results regarding all ITL variables. To evaluate practical significance, the SWC values for sRPE, LIT, MIT, and HIT were, respectively, 36 AU, 3.8 min, 1.4 min, and 1.3 min. Most of the comparisons exceed SWC and thus have practical value. A detailed description of the results is provided in the Supplementary Materials and Supplementary Table S3. A graphical representation is presented in Figure 4.

### 3.2. Between-Microcycles’ Internal Training Load Comparisons

The GEE analysis revealed a significant main effect of macrocycle on weekly sRPE, LIT, MIT, and HIT (all at *p* < 0.001). EMMs showed the highest ITL values during the preseason, followed by the in-season regular period and the in-season congested period. Within the in-season phase, regular weeks showed higher subjective and objective ITL values than congested weeks for sRPE and LIT (all at *p* < 0.001) and for MIT and HIT (all at *p* < 0.01) (Table 6). The sensitivity analysis using LMM confirmed these findings, except it did not converge for LIT variables, likely due to minimal between-player variability. In this case, the result relied primarily on the GEE results.

### 3.3. Validity, Agreement, and Reproducibility of Internal Training Load Measures

#### 3.3.1. Maximum Heart Rate

The athletes achieved 193 ± 4 bpm and 195 ± 4 bpm of their HRmax during the skating shuttle test (ICC = 0.98, 95% IC 0.89–0.99).

#### 3.3.2. HRmax Stability over Microcycles

The mixed-effects model estimated a mean bias of −1.38 bpm (*p* < 0.001; 95% CI: −1.93 to −0.84 bpm). The total standard deviation derived from the model was 1.59 bpm, resulting in 95% limits of agreement of −4.50 to +1.74 bpm (Bias ± 1.96 × SD). These results indicate that most match-derived and high-intensity training HRmax values fell within approximately ±3–4 bpm of the shuttle-based skating HRmax values.

#### 3.3.3. Association Between sRPE and HR-Derived ITL

At the group level, Spearman rho between sRPE and TRIMPmod was 0.767, *p* < 0.001 (95% CI 0.737–0.794). Further, across all individual players, sRPE showed a consistently very large and statistically significant association with the TRIMPmod, with Spearman’s rho values ranging from 0.739 to 0.822 and narrow 95% confidence intervals (Table 7).

#### 3.3.4. Reproducibility of sRPE Across Microcycles

During the preseason, reproducibility was generally moderate for LIT and MIT across most weekdays, whereas sRPE and HIT showed greater variability across sessions. Higher ICC values were observed in the middle of the microcycle (Table 8). During the regular in-season phase, sRPE and HR-derived measures demonstrated good to excellent reproducibility across most microcycle days, particularly from MD+3 to MD. Finally, in the congested in-season phase, sRPE and HR intensity zones showed generally good reproducibility across most session types, with particularly high ICC values observed on MDs.

## 4. Discussion

This is the first study to provide both subjective and objective quantification of ITL in an international-level senior RH team, comparing within and between three microcycle types (pre-season, in-season regular, and in-season congested). Our results demonstrate that during in-season regular and congested microcycles, MDs are the most demanding days of the week regarding MIT and HIT, whereas in the pre-season period, the highest subjective ITL is observed on Mondays, which is the training day that follows a period of complete rest. Across macrocycles, outfielders experienced their greatest ITL during the pre-season, followed by the in-season regular weeks, and the lowest loads occurred during in-season congested weeks.

### 4.1. Within-Microcycles’ Internal Training Load Comparisons

#### 4.1.1. Pre-Season Microcycles

Outfielders in the pre-season report very high subjective ITL across all days; however, the distribution followed a clear tapering pattern, with progressively lower sRPE values observed on later microcycles’ training days (see Table 3). These results align with those reported in a professional soccer team in which there is a higher TL on the first day of the pre-season’s weekly training session compared to the last day of the week [50]. Such a tapering structure may help prevent excessive fatigue accumulation by facilitating neuromuscular and metabolic recovery, while maintaining the adaptive stimulus induced by training sessions.

Interestingly, the goalkeeper’s load remained relatively stable across days, suggesting constant engagement during sessions, which is consistent with the unique positional demands of short-court team sports in which goalkeepers cannot be substituted as frequently as outfield players [5], and their state of permanent alertness and psychological stress reflect cognitive demands and situational pressure.

#### 4.1.2. In-Season Regular Microcycles

During the in-season regular microcycles, the load distribution of outfield players reflects patterns previously documented in professional RH [32,51], as well as in soccer and futsal, in which the training intensity is progressively reduced as the match approaches, with MD-1 consistently reported as the least demanding day of the week [9,52,53]. This ITL distribution plays a crucial role in promoting favourable physical and molecular adaptations, while avoiding non-functional responses such as excessive fatigue or overreaching. The highest HIT values were observed on MDs, which is possibly due to the team meeting its most important daygoals of the week. In line with this, Fernández et al. [54] showed that none of the drills typically prescribed during RH training can replicate the peak conditional demands of an official match. Accordingly, our SSGs were not able to elicit the same stress response observed on MDs.

For the goalkeeper, the highest absolute MIT and HIT values were also found on MDs, underlining the influence of attentional and psychological demands in shaping performance during competition. Notably, these levels were not reached during training in any type of microcycle analysed, echoing patterns previously reported for professional soccer goalkeepers [55].

##### In-Season Congested Microcycles

In congested microcycles, outfield players reported their highest ITLs during both MDs, reinforcing the load imposed by official competitions. Unlike in regular in-season weeks, in which MD-1 is reserved for tapering, in congested schedules, athletes still performed high-intensity work on these training days. One possible reason is that MD+2/-1 occurred after a full day off, explaining the high ITL. This pattern seems to resemble that of a professional basketball team, in which the highest TL from practice was found on MD-2, with a TL similar to match demands on the second MD-1 [8].

As with outfielders, the goalkeeper’s highest MIT and HIT values were found during MDs, confirming that match play remains the most demanding stimulus for this position across all microcycle types.

### 4.2. Between-Microcycles’ Internal Training Load Comparisons

As expected, pre-season microcycles of outfielders involved higher ITL values compared to both in-season weeks (*p* < 0.001), predominantly driven by multiple daily sessions. This structure mirrors reports from elite teams’ pre-seasons, such as soccer [53,56] and basketball [57,58], in which high training volumes are typically used to develop physical fitness before competitive demands escalate. In line with this long-term goal, the elevated pre-season ITLs observed in the present study likely reflect an intentional emphasis on global conditioning and neuromuscular preparation in the absence of immediate match pressure. Moreover, the progressive transition toward more regulated and tapered microcycles during the competitive season supports the notion that ITLs are strategically modulated to balance adaptation, recovery, and readiness for competition.

Our sRPE values during the pre-season aligned with those reported in some of the cited studies (4343 ± 329 AU in Jeong et al., 2011 [56]). However, other investigations have shown considerably higher loads (e.g., ~5000 AU in a professional basketball team [58]), likely reflecting differences in weekly programming. On the contrary, regarding the time in MIT and HIT, the values of our team exceed those of a younger soccer team [43]. This difference could reflect both the lower training intensity associated with the players’ younger age and the distinct metabolic demands between soccer [59,60] and RH. The former, played on a larger pitch, typically involves a lower density of high-intensity actions, resulting in a reduced time in MIT and HIT responses.

Interestingly, in-season sRPE values of our regular weeks exceeded those reported in soccer (1703 ± 173 AU in Jeong et al. [56]). This may be explained by the fact that this RH team trained more than the reported soccer squad (average weekly training exposure: 101 ± 32 min vs. 83 ± 15 min in Jeong et al. [56]). A higher training stimuli during the in-season period, as reported in our investigation, could potentially mitigate the risk of performance decline observed in soccer teams [61]; however, this is just a hypothesis, as we have not measured their physical performance modifications across the season. Additionally, our MIT and HIT minutes seem aligned with those monitored in futsal during a month of an in-season microcycle (one match/week), despite this team being composed of amateur male practitioners [62]. This comparison seems to highlight the similarity in metabolic demands between RH [3] and futsal [63]. This resemblance likely stems from the shared intermittent profile of both sports performed on a similar indoor pitch, which requires repeated bouts of high-intensity actions interspersed with brief recovery periods [63].

Moreover, our analysis revealed that in-season regular weeks were characterised by higher ITL (sRPE, LIT, MIT) but similar HIT values compared to congested microcycles (Table 6). This contrasts with findings in soccer, in which the weekly sRPE values remained similar between regular and congested weeks [64]. Notably, the absolute values reported in that study (~1500 AU in regular weeks and ~1800 AU in congested weeks [64]) are comparable to our congested values, suggesting that the TL of that soccer team during in-season regular weeks may be underdosed.

Conversely, a professional high-level basketball team reported comparable ITL values between in-season regular and congested weeks (~2800 AU [58]). Notably, their congested-week ITL was comparable to that observed in our in-season regular microcycles. This may be explained by their higher pre-season training loads, which could have enabled them to maintain elevated ITL values throughout the season, along with the inclusion of an additional training day, compared to the investigated RH team, during congested periods. Notably, the comparison with a basketball team is particularly informative at a macro-organisational level, as the two sports share several contextual characteristics, including a relatively small playing court, repeated high-intensity efforts interspersed with brief recovery periods, and a stop-clock system during matches [65]. Accordingly, similar HR responses during competition have been reported in basketball [65,66], suggesting comparable global cardiovascular demands [3,65]. From this perspective, RH teams may consider adopting elements of basketball-based training organisation, such as a higher training frequency, to support competitive readiness.

However, despite these similarities, RH presents sport-specific locomotor and mechanical demands that may require distinct ITLs. In particular, skating-based locomotion is characterised by lower vertical oscillation and a distinct force–velocity relationship compared with running [67,68,69], and the frequent accelerations and directional changes typical of RH may increase glycolytic and neuromuscular demands that are not fully captured by HR-based metrics alone. Therefore, to avoid excessive neuromuscular fatigue, particularly in the presence of frequent accelerations and decelerations, the training volume, especially skating-based high-intensity work, may need to be more carefully regulated. Finally, the limited time spent in HIT observed in the present study during training suggests that commonly used SSG formats may be less effective in eliciting match-equivalent HIT. Collectively, these sport-specific characteristics highlight the need for tailored microcycle structures and caution when extrapolating internal training-load distribution models from other intermittent team sports.

### 4.3. Agreement and Reproducibility of Internal Training Load Measures

The agreement between HRmax values derived from MDs and high-intensity training showed a small and consistent bias relative to the skating-based HRmax test, with narrow limits of agreement, indicating stable maximal cardiovascular responses across microcycles.

In addition, sRPE demonstrated a very large and consistent association with objective ITLs at both the group and individual levels, supporting its validity as a marker of perceived training stress. This finding adds up to previous research in RH, which has reported significant and positive associations between sRPE and external load indicators, suggesting that sRPE is sensitive to variations in training demands [32,51] and, ultimately, supporting its use for monitoring weekly TL.

Reproducibility analyses further showed that sRPE and HR-based intensity zones exhibited moderate to excellent reliability across microcycles, particularly during the regular and congested in-season phases, especially for HIT. Although this is the first study to investigate these relationships in RH, the present findings are consistent with previous research reporting strong associations between subjective ITL and HR-derived ITLs in intermittent team sports [70].

Collectively, these findings indicate that sRPE and HR-derived metrics provide a robust and internally consistent representation of TL distribution and may be useful for monitoring TL in RH.

## 5. Practical Application

This investigation may provide preliminary evidence-based insights to guide the management of ITL distribution within an international-level RH team across a competitive season. Overall, the findings suggest that ITL should be progressively reduced as MD approaches and that the higher overall ITL could be implemented following a rest day and/or at the greatest temporal distance from competitions. Finally, pre-season microcycles may, and ideally should, present higher weekly ITL values compared to in-season periods, to promote the development of physical conditioning and team play.

## 6. Limitations and Future Directions

The restricted cohort of international-level athletes that has been analysed, reflecting the small number of players that typically comprise an RH team (eight outfielders and two goalkeepers), and the absence of a priori sample size analysis do not allow us to draw broader inferences to matched-level players. This constraint is inherent to research conducted in elite team-sport settings, which usually recruits a convenience sample. Further investigations on TL monitoring are warranted in other senior winning teams to confirm or refute the efficiency of the periodisation and training strategies adopted by the team throughout the season.

Another limitation may be the absence of a retest of the skating shuttle test; however, this is mitigated by the high agreement between the HRmax obtained during the shuttle test and the HRmax recorded during all matches and a weekly training session.

In addition, monitoring ITL alongside external TL, such as that obtained from inertial sensors, would provide valuable insights into the positional demands of RH athletes, enabling the creation of SSGs with TL demands similar to MDs. Additionally, the absence of ETLs may have influenced the interpretation of training load distribution, as HR-based metrics are known to plateau at high intensities and may underestimate short-duration, high-intensity mechanical demands. Consequently, some neuromuscular or locomotor loads typical of HR may not be fully captured by HR-derived ITL alone.

Furthermore, this analysis did not account for contextual variables, which have been considered in previous studies in this sport. Incorporating factors such as match location (home/away) or opponent difficulty (easy/medium/difficult) into the statistical model would have generated a larger number of microcycle types, thereby reducing the statistical power of the analysis. Hence, future studies spanning several monitoring seasons should consider these variables in order to provide a better understanding of how TL is distributed and, consequently, to inform specific training guidelines.

Another major limitation is the lack of domain-specific fatigue assessment (e.g., neuromuscular, mental, etc.). Such measures could have provided valuable information to better contextualise athletes’ day-to-day readiness and to determine whether the prescribed ITL for each training session was appropriately tolerated and, thus, effectively planned.

Finally, only one goalkeeper’s data was included, as the second goalkeeper, being of a lower technical level, did not consistently participate in matches except when temporarily substituting for the first. This resulted in very different ITL measurements between the two athletes. Therefore, future studies should include goalkeepers from multiple teams in order to obtain role-specific data, which could then be used to characterise position-specific demands.

## 7. Conclusions

An international-level RH team progressively tapered ITLs across later training days within pre-season microcycles and as MDs approached during the regular season. Accordingly, the highest overall ITL was typically scheduled after a rest day in the pre-season or at the greatest temporal distance from MDs during the in-season regular microcycle. Finally, pre-season ITLs were higher than those reported during in-season microcycles, which, in turn, exhibited greater ITLs than those observed during congested in-season weeks. This ITL distribution may be of relevance for RH coaches and support staff aiming to optimise microcycle planning across different phases of the competitive season.

## Figures and Tables

**Table 1:** Detailed description of training days according to macrocycles.

Macrocycles	Training, Duration	Description
Preseason	Mon 200 ± 24 min	Morning: 15–25 min of introductory technique-activation exercises (INT), followed by 65–90 min of analytical situations (ANLS). A 10 min break was included in the middle of the session. Evening: 10–20 min of INT, followed by 15–30 min of ANLS, and full-court SSGs (4vs4, 40 × 20 m in different formats, such as 4 sets × 4 min up to 2–3 sets × 10 min, 3–5 min of recovery between sets). A 10 min break was included in the middle of the session.
	Tue 196 ± 21 min	Morning: 15–25 min of INT, followed by 65–90 min of ANLS. A 10 min break was included in the middle of the session. Evening: 10–20 min of INT, followed by 15–30 min of ANLS, and by mid-court SSGs (4vs4, 20 × 20 m, from 2 sets of 4 min to 4 sets of 4 min) and/or full-court superiorities situations (3vs4, 40 × 20 m, from 2 sets of 2 min to 4 × 2 min, 2–3 min of recovery between sets). A 10 min break was included in the middle of the session.
	Wed 158 ± 18 min	Morning: 15–25 min of INT, followed by 30–45 min of ANLS. A 10 min break was included in the middle of the session. Evening: 10–20 min of INT, followed by 15–30 min of ANLS, and full-court SSGs (4vs4, 40 × 20 m, such as 2 sets × 4 min, 3 min of recovery between sets). A 10 min break was included in the middle of the session.
	Thu 164 ± 43 min	Morning: 15–25 min of INT, followed by 40–60 min of ANLS. A 10 min break was included in the middle of the session. Evening: 10–20 min of INT, followed by 15–30 min of ANLS, and by mid-court SSGs (4vs4, 20 × 20 m, from 2 sets of 4 min to 4 sets of 4 min, 3 min of recovery between sets) and/or full-court superiorities situations (3vs4, 40 × 20 m, from 2 sets of 2 min to 4 × 2 min).
	Fri 115 ± 37 min	Morning: 15–25 min of INT, followed by 30–45 min of ANLS. Evening: 10–20 min of INT, followed by 15–30 min of ANLS, and by mid-court SSGs (4vs4, 20 × 20 m, from 2 sets of 4 min to 4 sets of 4 min, 3 min of recovery between sets). No break within these short sessions.
	Sat 74 ± 4 min	Replication of match characteristics (two halves of 25 min of RH practice with min official game time). Recovery of 10 min between halves.
Regular In-season	MD+2 131 ± 51 min	Morning: 15–25 min of INT, followed by 30–60 min of ANLS. Evenings: 10–20 min of INT, followed by 30–45 min of ANLS, and mid-court SSGs (e.g., 4vs4, 20 × 20 m, 4 × 4 min, 2–3 min breaks in between).
	MD+3 149 ± 40 min	Morning: 15–25 min of INT, followed by 30–60 min of ANLS. Evenings: 10–20 min of INT, followed by match-replication drills (4vs4, 40 × 20 m, e.g., 4 sets × 10 min, with 3–5 min breaks in between).
	MD-3 95 ± 18 min	Afternoon: 15–25 min of INT, followed by 30–45 min of ANLS, and mid-court SSGs (e.g., 4vs4, 20 × 20 m, ranging from 2 × 4 min to 4 × 4 min, 3 min breaks in between). A 10 min break was included in the middle of the session.
	MD-2 98 ± -21 min	Afternoon: 15–25 min of INT, followed by 30–45 min of ANLS, followed by match-replication (4vs4, 40 × 20 m, e.g., 4 sets × 10 min, with 3–5 min breaks in between). A 10 min break was included in the middle of the session.
	MD-1 69 ± 14 min	Afternoon: 15–25 min of INT, followed by mid-court blue-card situations, individual scoring to the goalposts, and relaxed team games.
	MD 75 ± 7 min	Official matches
Congested In-season	MD+2/-2 90 ± 19 min	Afternoon: 15–25 min of INT, followed by 30–45 min of ANLS, and by mid-court SSGs (e.g., 4vs4, 20 × 20 m, 4 × 4 min, 3 min breaks in between). A 10 min break was included in the middle of the session.
	MD-1 67 ± 8	Afternoon: 15–25 min of INT, followed by a full-court SSGs (4vs4, 40 × 20 m, from 2 sets × 4 min to 4 sets × 2 min, 1–2 min of break), followed by mid-court blue-card situations, individual scoring to the goalposts, and relaxed team games
	MD1 75 ± 4	Official matches
	MD+2/-1 96 ± 15	Afternoon: 15–25 min of INT, followed by 30–45 min of ANLS and by full-court SSGs (e.g., 4vs4, 40 × 20 m, 4 × 4 min, 3 min breaks in between). A 10 min break was included in the middle.
	MD2 77 ± 4	Official matches

INT: introductory technique-activation exercises, such as skating activation with changes in direction, manipulating the stick with the ball, and associating passes with/without opposition. 1 × 1, etc. ANLS: analytical situations, such as analytical, tactical movements with/without opponents with passes, specific changes in direction, and finalisation (i.e., shooting), changes in position while skating and passing, finishing with a shot on goal from different positions, etc. SSGs: small-sided games.

**Table 2:** Demographic, anthropometric, physiological, and professional characteristics of athletes.

ID	Age (Years)	Body Mass (kg)	Height (m)	HRmax (bpm)	Position, Role	Professional RH Practice (Years)	National Team RH Practice (Years)	Championship		
								National	European	World
1	23	74.5	179.4	197	FOR	8	7	2	1 gold	
2	33	83.2	176.4	187	FOR	14	0			
3	23	69.3	176.8	198	FOR	8	0			
4	32	83.6	180.4	196	GK	18	14	3		2 gold, 5 silver, 2 bronze
5	32	86.7	183.5	198	DEF	18	14	2	1 gold, 1 silver, 3 bronze	
6	33	75.4	178.2	191	FOR	19	0			
7	34	78.6	179.5	193	DEF	19	0			
8	23	80.3	180.7	197	DEF	8	0			

**Table 3:** Descriptive data of sRPE, LIT, MIT, and HIT for outfielders and the goalkeeper during the pre-season microcycles.

Position	Variables	Monday (a)	Tuesday (b)	Wednesday (c)	Thursday (d)	Friday (e)	Saturday (f)
Outfielders	sRPE (AU)	807 ± 40 (726–886) b †, c †, d †, e #, f †	643 ± 13 (615–659) a †, c †, e †, f †	705 ± 24 (658–752) a †, b †, e †, f †	651 ± 42 (569–732) a †, e †, f †	552 ± 34 (484–618) a †, b †, c †, d †	534 ± 11 (511–555) all †
	LIT (min)	158 ± 3 (152–163) all †	175 ± 2 (172–177) All †	122 ± 2 (117–126) All †	134 ± 2 (129–139) all †	93 ± 1 (91–96) all †	41 ± 2 (37–45) all †
	MIT (min)	29 ± 3 (24–34) b †, c *, d †, e †, f †	13 ± 1 (11–15) a †, c *, d †, f *	24 ± 2 (20–29) a *, b †, d †, e †, f *	20 ± 2 (17–23) a †, b †, c †, e †	13 ± 2 (9–16) a †, c †, d †	17 ± 2 (13–20) a †, b *, c *, e *
	HIT (min)	13 ± 1 (11–15) a †, e †	8 ± 1 (7–10) a †, c †, f †	12 ± 1 (10–13) b †, e *, s †	10 ±1 (7–13) f *	9 ± 1 (8–10) a †, c #, f †	16 ± 1 (13–19) b †, c †, d #, e †
Goalkeeper	sRPE (AU)	769 ± 106 (371–826)	604 ± 106 (376–831)	694 ± 106 (466–921)	599 ± 106 (371–826)	490 ± 106 (262–717)	537 ± 106 (308–764)
	LIT (min)	155 ± 23 (106–204)	173 ± 23 (123–222)	129 ± 23 (79–177)	143 ± 23 (93–191)	89 ± 23 (40–138)	41 ± 23 (17–91)
	MIT (min)	29 ± 6 (16–43)	14 ± 6 (8–27)	19 ± 6 (5–32)	14 ± 6. (8–27)	21 ± 6 (8–35)	20 ± 6 (6–34)
	HIT (min)	15 ± 2 (11–19)	9 ± 2 (5–14)	10 ± 2 (6–14)	7 ± 2 (3–12)	4 ± 2 (1–9)	12 ± 2 (8–17)

Data are presented as EMMs ± SE (95% CI, lower–upper). AU = arbitrary units; min = minutes; sRPE = session rating of perceived exertion; LIT = low-intensity training; MIT = medium-intensity training; HIT = high-intensity training. Letters indicate significant differences between days. Symbols: * *p* < 0.05, # *p* < 0.01, † *p* < 0.001.

**Table 4:** Descriptive data of sRPE, LIT, MIT, and HIT for outfielders and the goalkeeper during the in-season regular microcycles.

Position	Variables	MD+2 (a)	MD+3 (b)	MD-3 (c)	MD-2 (d)	MD-1 (e)	MD (f)
Outfielders	sRPE (AU)	499 ± 28 (450–561) b †, c †, e †	747 ± 40 (659–824) all †	401 ± 31 (341–361) all †	526 ± 27 (473–578) b †, c †, e †	141 ± 17 (107–174) all †	528 ± 11 (506–550) b †, c †, e †
	LIT (min)	114 ± 1 (111–116) all †	125 ± 2 (122–129) all †	79 ± 1 (77–82) all †	63 ± 1 (61–65) all †	67.5 ± 0 (67–67) all †	42 ± 2 (39–46) all †
	MIT (min)	10 ± 1 (8–12) b †, d †, e †, f †	19 ± 1 (17–22) a †, c †, e †, f *	11 ± 1 (9–13) b †, d †, e †, f †	20 ± 1 (18–21) a †, c †, e †, f †	0 ± 0 (n.a.) all †	17 ± 2 (15–19) a †, b *, c †, d #, e †
	HIT (min)	3 ± 1 (2–4) all †	9 ± 1 (7–11) a †, c †, e †, f †	4 ± 1 (3–6) all †	10 ± 1 (9–11) a †, c †, e †, f †	0 ± 0 (n.a.) all †	17 ± 2 (14–20) all †
Goalkeeper	sRPE (AU)	530 ± 74 (428–725)	834 ± 74 (684–982)	346 ± 74 (161–457)	418 ± 74 (269–564)	116 ± 74 (85–264)	488 ± 74 (339–636)
	LIT (min)	117 ± 7 (103–130)	129 ± 7 (115–142)	84 ± 7 (70–97)	83 ± 7 (69–96)	67 ± 7 (54–80)	35 ± 7 (22–49)
	MIT (min)	7 ± 2 (3–11)	17 ± 2 (13–21)	8 ± 2 (4–12)	7 ± 2 (3–11)	0 ± 0 (n.a.)	29 ± 2 (29–36)
	HIT (min)	2 ± 2 (1–5)	8 ± 2 (5–10)	2 ± 2 (1–5)	3 ± 2 (1–5)	0 ± 0 (n.a.)	11 ± 2 (9–14)

Data are presented as EMMs ± SE (95% CI, lower–upper). AU = arbitrary units; min = minutes; sRPE = session rating of perceived exertion; LIT = low-intensity training; MIT = medium-intensity training; HIT = high-intensity training. MD ± X = relative day from match (MD on Saturday); n.a. = not applicable. Letters indicate significant differences between training days. Symbols: * *p* < 0.05, # *p* < 0.01, † *p* < 0.001.

**Table 5:** Descriptive data of sRPE, LIT, MIT, and HIT for outfielders and the goalkeeper during the in-season congested microcycles of the National Championship.

Position	Variables	MD+2/-2 (a)	MD-1 (b)	MD1 (c)	MD+2/-1 (d)	MD2 (e)
Outfielders	sRPE (AU)	358 ± 38 (282–432) b †, c †, e *	175 ± 14 (146–202) all †	492 ± 22 (447–536) a #, b #, d †	352 ± 28 (297–407) b #, c #, e †	465 ± 25 (416–513) a #, b †
	LIT (min)	68 ± 2 (64–73) all †	55 ± 2 (53–58) all †	42 ± 2 (39–46) all †	80 ± 2 (76–83) all †	46 ± 2 (42–50) all †
	MIT (min)	15 ± 2 (12–18) b †, d #	8 ± 1 (6–10) all †	16 ± 1 (14–18) b †, d †	12 ± 1 (10–14) b #, c †	13 ± 1 (11–15) b †
	HIT (min)	6 ± 1 (5–8) b #, c †, d #, e †	4 ± 1 (2–5) a †, c †, e †	17 ± 2 (14–20) a †, b †, d †	4 ± 1 (2–6) a #, c †, e †	17 ± 2 (14–20) a †, b †, d †
Goalkeeper	sRPE (A.U.)	228 ± 62 (99–357)	189 ± 62 (69–318)	415 ± 62 (285–544)	382 ± 62 (252–511)	459 ± 62 (329–587)
	LIT (min)	80 ± 7 (65–94)	55 ± 7 (41–69)	36 ± 7 (22–51)	91 ± 7 (77–105)	39 ± 7 (24–53)
	MIT (min)	7 ± 3 (1–12)	9 ± 3 (3–14)	28 ± 3 (22–34)	4 ± 3 (2–10)	29 ± 3 (22–34)
	HIT (min)	3 ± 2 (1–7)	3 ± 2 (1–7)	11 ± 2 (7–14)	1 ± 2 (−2–4)	9 ± 2 (6–13)

Data are presented as EMMs ± SE (95% CI, lower–upper). AU = arbitrary units; min = minutes; sRPE = session rating of perceived exertion; LIT = low-intensity training; MIT = medium-intensity training; HIT = high-intensity training. MD ± X = relative day from match; congested schedule contains two matches per week (MD1 on Wednesday and MD2 on Saturday). Letters indicate significant differences between training days. Symbols: * *p* < 0.05, # *p* < 0.01, † *p* < 0.001.

**Table 6:** Outfielders’ between-microcycles’ internal training load comparisons.

Microcycles	sRPE (AU)	LIT (min)	MIT (min)	HIT (min)
Pre-season (a)	3891 ± 141 (3614–4167) b †, c †	722 ± 7 (707–737) b †, c †	116 ± 8 (100–131) b †, c †	67 ± 3 (60–75) b †, c †
In-season regular (b)	2850 ± 137 (2579–3119) a †, c †	492 ± 5 (481–502) a †, c †	77 ± 4 (68–86) a †, c #	47 ± 3 (40–54) a †, c #
In-season congested (c)	1842 ± 89 (1665–2017) a †, b †	292 ± 6 (280–304) a †, b †	65 ± 4 (56–73) a †, b #	43 ± 2 (38–48) a †, b #

Data are presented as EMMs ± SE (95% CI, lower–upper). AU = arbitrary units; min = minutes; sRPE = session rating of perceived exertion; LIT = low-intensity training; MIT = medium-intensity training; HIT = high-intensity training. Letters indicate significant differences between microcycle types. Symbols: # *p* < 0.01, † *p* < 0.001.

**Table 7:** Within-subject (player-level) associations.

ID	Spearman Rho	*p* Value	95% CI
1	0.784	<0.001	0.702–0.846
2	0.779	<0.001	0.695–0.842
3	0.821	<0.001	0.751–0.873
4	0.755	<0.001	0.650–0.831
5	0.822	<0.001	0.752–0.874
6	0.791	<0.001	0.710–0.851
7	0.739	<0.001	0.643–0.813
8	0.782	<0.001	0.699–0.845

Significance level *p* < 0.05; CI: confidence intervals.

**Table 8:** Reproducibility of ITL values across macrocycles.

Variable	N° Weeks	Macrocycle									
		Preseason									
		Mon	Tue		Wed		Thu		Fri		Sat
sRPE	4	0.38 (−0.84–0.86)	0.44 (−0.25–0.68)		0.53 (−0.19–0.83)		0.28 (−1.15–0.84)		0.28 (−1.14–0.84)		0.60 (0.23–0.79)
LIT	4	0.55 (−0.32–0.90)	0.52 (−0.41–0.89)		0.62 (−0.12–0.91)		0.66 (−0.02–0.92)		0.14 (−1.56–0.80)		0.40 (−0.79–0.86)
MIT	4	0.64 (−0.07–0.92)	0.50 (−0.50–0.89)		0.72 (0.16–0.94)		0.59 (−0.22–0.90)		0.34 (−0.95–0.85)		0.44 (−0.67–0.87)
HIT	4	0.11 (−1.65–0.80)	0.54 (−0.36–0.90)		0.58 (−0.25–0.91)		0.79 (0.37–0.95)		0.25 (−1.22–0.83)		0.71 (0.14–0.94)
		Regular In-season									
		MD+2	MD+3		MD-3		MD-2		MD-1		MD
sRPE	12	0.55 (−0.15–0.89)	0.82 (0.54–0.94)		0.73 (0.31–0.98)		0.89 (0.71–0.97)		0.83 (0.57–0.96)		0.61 (0.03–0.91)
LIT	12	0.62 (0.03–0.91)	0.72 (0.27–0.93)		0.76 (0.40–0.94)		0.91 (0.77–0.98)		n.a.		0.76 (0.39–0.94)
MIT	12	0.66 (0.14–0.92)	0.74 (0.33–0.94)		0.65 (0.01–0.92)		0.83 (0.55–0.96)		n.a.		0.86 (0.65–0.97)
HIT	12	0.44 (−0.44–0.87)	0.61 (−0.00–0.91)		0.75 (0.38–0.94)		0.84 (0.60–0.96)		n.a.		0.82 (0.54–0.96)
		Congested In-season									
		MD+2/-2		MD-1		MD		MD+2/-1		MD	
sRPE	5	0.88 (0.67–0.97)		0.35 (−0.81–0.85)		0.73 (0.24–0.94)		0.70 (0.37–0.90)		0.60 (−0.11–0.91)	
LIT	5	0.78 (0.40–0.95)		0.54 (−0.27–0.90)		0.67 (0.08–0.93)		0.67 (0.08–0.93)		0.83 (0.54–0.96)	
MIT	5	0.73 (0.27–0.95)		0.51 (−0.36–0.89)		0.78 (0.39–0.95)		0.53 (−0.31–0.89)		0.91 (0.75–0.98)	
HIT	5	0.79 (0.40–0.95)		0.26 (−1.06–0.83)		0.79 (0.42–0.95)		0.77 (0.36–0.94)		0.90 (0.73–0.98)	

sRPE = session rating of perceived exertion; LIT = low-intensity training; MIT = medium-intensity training; HIT = high-intensity training.

## Data Availability

The data associated with the paper are not publicly available but are available from the corresponding author (G.D.) upon reasonable request.

## Supplementary Materials

The following supporting information can be downloaded at: https://www.mdpi.com/article/10.3390/jfmk11010016/s1. Results of the Generalised Estimating Equations (GEE).
