# Supplementary material for: Training Load Distribution Across Weekly Microcycles According to the Match Schedule During the Regular Season in a Professional Rink Hockey Team
Source: J Funct Morphol Kinesiol. 2025 Dec 29;11(1):16. doi: 10.3390/jfmk11010016 (PMC12821721; doi:10.3390/jfmk11010016)
Supplement: Supplementary file 1 [file jfmk-11-00016-s001.zip › jfmk-3976475-supplementary.pdf]

**Results of the Generalised Estimating Equations (GEE)**

***Within-microcycle comparison***

*Pre-season microcyles*

Session rating of perceived exertion (sRPE)

The GEE model revealed significant effects of microcycle (Wald $\chi^2(3)=194.08$, p<0.001), training day (Wald $\chi^2(5)=2198.19$, p<0.001), and their interaction (Wald $\chi^2(7)=4.84 \times 10^{11}$, p<0.001). Across microcycles, sRPE was reduced in the fourth week compared to the first (p=,022) and second (p<0.001), with the peak value on the second week (statistically significant vs all). Estimated marginal means (EMMs) showed that sRPE increased from the first to the second microcycle (658,92±53,91 AU to 770,53±30,19 AU) and then decreased progressively to the fourth. sRPE varied significantly across days. The highest values were observed on Monday (806.71±40.81 AU) and Wednesday (705.46±23.98 AU), while the lowest occurred on Saturday (533.73±11.31 AU) and Friday (551.50±34.16 AU) (Table S1). Most pairwise differences were significant (from p<0.05 to p<0.001). Significant interactions indicated that changes in sRPE across days depended on the microcycle. For instance, the fourth microcycle showed strong decreases on later days (e.g., Friday: −614.14±71.45 AU, p<0.001), whereas the central weeks showed mixed patterns with both increases and decreases across days.

The sensitivity analysis using Linear Mixed Model (LMM) confirmed these findings, showing a significant fixed effect of microcycle (F(3,138)= 17,254, p < 0.001), training days (F(5,138)= 12,715, p < 0.001), as well as their interaction (F(7,138)= 15,066, p < 0.001). Pairwise comparisons reproduced the same pattern observed in the GEE analysis. The model demonstrated large effect sizes, with preseason weeks explaining a meaningful proportion of the variance ($R^2$ marginal = 0.638), while the inclusion of random effects increased the explained variance ($R^2$ conditional = 0.688).

*Low-Intensity training (LIT)*

The GEE model revealed significant effects of microcycle (Wald $\chi^2(3)=3252.51$, p<0.001), training day (Wald $\chi^2(5)=50390.88$, p<0.001), and their interaction (Wald $\chi^2(7)=9.06 \times 10^{14}$<0.001). Significant differences across microcycles were observed. EMMs showed a progressive reduction in LIT load across the four preseason microcycles, with values decreasing from microcycle 1 (146.98 ± 1.62 min) and microcycle 2 (143.27 ± 1.40 min) to microcycle 3 (101.26 ± 1.39 min) and microcycle 4 (90.04 ± 1.51 min). All pairwise comparisons between microcycles were statistically significant (p < 0.001), confirming significant reductions between all microcycles. Daily LIT varied significantly, with peaks on Tuesday (174.82±1.32 min) and Monday (157.59±2.79 min), and minima on Saturday (41.04±2.26 min) (Table S1). Pairwise comparisons were all significant (p<0.001). The interaction was significant, indicating that daily LIT volume depended on the microcycle. Specifically, central weeks displayed mixed increases and decreases across days.

The sensitivity analysis using LMM confirmed these findings, showing a significant fixed effect of microcycle (F(3,138)= 480.894, p < 0.001), training days (F(5,138)= 81,972, p < 0.001), as well as their interaction (F(7,138)= 173,503, p < 0.001). Pairwise comparisons reproduced the same pattern observed in the GEE analysis. The fixed effects explained almost all the variance in LIT ($R^2$ marginal = 0.978), while the inclusion of random effects very slightly increased the explained variance ($R^2$ conditional = 0.981).

Moderate-Intensity training (MIT)

The GEE model identified significant main effects of microcycle (Wald $\chi^2(3)=62.52$, p<0.001), weekday (Wald $\chi^2(5)=600.87$, p<0.001), and their interaction (Wald $\chi^2(7)=7.87 \times 10^{12}$, p<0.001). MIT differed significantly across microcycles. EMMs were highest in the second (22.33±1.36 min) and lowest in the fourth (15.81±1.25 min). Pairwise comparisons revealed significant differences between all microcycles (from p < 0.05 to p<0.001).

Daily MIT varied significantly. Peaks occurred on Monday (29.00±2.72 min) and Wednesday (24.39±2.08 min), with minima on Friday (12.61±1.57 min) (Table S1). Pairwise comparisons confirmed that most daily differences were significant (from $p<0.05$ to $p<0.001$). The significant interaction indicates that daily MIT depended on the microcycle. For example: the fourth week showed substantial decreases on Saturday (−36.14±5.36 min, $p < 0.001$) and Wednesday (−26.57±5.64 min, $p < 0.001$), with smaller changes on other days, while the central weeks displayed mixed increases and decreases across days,

The sensitivity analysis using LMM confirmed these findings, showing a significant fixed effect of microcycle ($F(3,138)= 7.764$, $p < 0.001$), training days ($F(5,138)= 26.816$, $p < 0.001$), as well as their interaction ($F(7,138)= 10.926$, $p < 0.001$). Pairwise comparisons reproduced the same pattern observed in the GEE analysis. The model demonstrated large effect sizes, with preseason weeks explaining a meaningful proportion of the variance ($R^2$ marginal = 0.599), while the inclusion of random effects increased the explained variance ($R^2$ conditional = 0.689).

High-Intensity training (HIT)

The GEE models revealed significant main effects of microcycle (Wald $\chi^2(3)=259.86$, $p<0.001$), weekday (Wald $\chi^2(5)=222.35$, $p<0.001$), and their interaction (Wald $\chi^2(7)=6{,}4 \times 10^14$, $p<0.001$). HIT exposure differed significantly across microcycles. EMMs were highest in the first week (14.71±0.55 min) and statistically significant versus all others ($p<0.001$). The lowest value, measured in the second microcycle, was statistically significant compared to the first and the third week (both $p<0.001$). Daily HIT exposure varied significantly, with peaks on Saturday (16.07±1.45 min) and Monday (13.16±0.88 min), and minima on Friday (8.79±0.44 min) and Tuesday (8.32±0.72 min) (Table S1). Pairwise comparisons indicated significant differences for most day-to-day comparisons ($p < 0.05$). The significant interaction shows that HIT exposure was microcycle-specific across days: the third week showed mixed increases and decreases across days, with a notable increase on Saturday (+7.57±2.31 min, $p=0.001$), while the second microcycle displayed minimal day-to-day variation.

The sensitivity analysis using LMM confirmed these findings, showing a significant fixed effect of microcycle ($F(3,138)= 14{,}876$, $p < 0.001$), training days ($F(5,138)= 15{,}205$, $p < 0.001$), as well as their interaction ($F(7,138)= 15{,}363$, $p < 0.001$). Pairwise comparisons reproduced the same pattern observed in the GEE analysis. The model demonstrated large effect sizes, with preseason weeks explaining a meaningful proportion of the variance ($R^2$ marginal = 0.644), while the inclusion of random effects increased the explained variance ($R^2$ conditional = 0.693).

Table S1. Pairwise comparisons of pre-season within-microcycle training-day internal load variables in outfield players

| Position | Variable | Training days | Pairwise comparison |
|---|---|---|---|
| **Outfielders** | | | |
| | **sRPE (AU)** | | |
| | | **Mon** | Tue (< .001), Wed (< .001), Thu (< .001), Fri (.010), and Sat (< .001) |
| | | **Tue** | Mon (< .001), Wed (< .001), Fri (.001), Sat (< .001), |
| | | **Wed** | Mon (< .001), Tue (< .001), Fri (< .001), and Sat (< .001) |
| | | **Thu** | Mon (< .001), Fri (< .001), and Sat (.001) |
| | | **Fri** | Mon (< .001), Tue (.001), Wed (< .001), and Thu (< .001) |
| | | **Sat** | Mon (< .001), Tue (< .001), Wed (< .001), Thu (<0.001), and Fri (.001) |
| | **LIT (min)** | | |

| | | |
|---|---|---|
| | **Mon** | vs all (<0.001) |
| | **Tue** | vs all (<0.001) |
| | **Wed** | vs all (<0.001) |
| | **Thu** | vs all (<0.001) |
| | **Fri** | vs all (<0.001) |
| | **Sat** | vs all (<0.001) |
| **MIT (min)** | | |
| | **Mon** | Tue (< .001), Wed (.031), Thu (< .001), Fri (< .001), and Sat (< .001) |
| | **Tue** | Mon (< .001), Wed (< .001), Thu (< .001), Sat (.032) |
| | **Wed** | Mon (.031), Tue (< .001), Thu (< .001), Fri (< .001), and Sat (0.013) |
| | **Thu** | Mon (< .001), Tue (< .001), Wed (< .001), Fri (< .001) |
| | **Fri** | Mon (< .001), Wed (< .001), and Thu (< .001) |
| | **Sat** | Mon (< .001),Tue (.032), Wed (.013), and Fri (.044) |
| **HIT (min)** | | |
| | **Mon** | Tue (<.001) and Fri (< .001) |
| | **Tue** | Mon (<.001), Wed (< .001) and Sat (<.001) |
| | **Wed** | Tue (< .001), Fri (.003), and Sat (< .001) |
| | **Thu** | Sat (0.002) |
| | **Fri** | Mon (< .001), Wed (.003), and Sat (< .001) |
| | **Sat** | Tue (<.001), Wed (< .001), Thu (.002), and Fri (<.001) |

Mon = Monday; Tue = Tuesday; Wed = Wednesday; Thu = Thursday; Fri = Friday; Sat = Saturday; AU = arbitrary units; min = minutes; sRPE = session rating of perceived exertion; LIT = low-intensity training; MIT = medium-intensity training; HIT = high-intensity training; n.s. = not significant.

*Regular In-season microcycles*

Session rating of perceived exertion (sRPE)

The GEE models revealed significant main effects of microcycle (Wald $\chi^2(7)$=4,7× $10^{1}$1, p<0.001), training days (Wald $\chi^2(5)$=9435,78, p<0.001), and their interaction (Wald $\chi^2(8)$=1,3 × $10^{1}$4, p<0.001) indicating that the distribution of internal load across the microcycle varied substantially across different in-season weeks. EMMs showed marked week-to-week fluctuations in sRPE, with higher mean values observed in specific weeks (e.g., weeks 2 and 8) compared with others (e.g., weeks 7, 11, and 12), while within the microcycle, sRPE was systematically lower on days MD−1 and MD−3 and substantially higher on MD+2 and MD+3 (Table S2). The significant interaction highlights that these daily loading patterns were not consistent across weeks, reflecting dynamic weekly modulation of training load during the in-season phase.

The LMM confirmed the GEE results, showing significant fixed effects of week ($F(11,426)$= 17.28, $p < 0.001$), training day ($F(5,426)$= 260.37, $p < 0.001$), and a significant week × day interaction ($F(55,426)$= 10.72, $p < 0.001$). The model demonstrated a high explanatory capacity, with a marginal $R^2$ of 0.758 and a conditional $R^2$ of 0.817, indicating that fixed effects accounted for a large proportion of the variance in sRPE.

Low-intensity training (LIT)

The GEE revealed a significant main effect of in-season week (Wald $\chi^2(9)$= 5.20 × $10^{13}$, $p < 0.001$), a significant main effect of training day (Wald $\chi^2(5)$= 5050.72, $p < 0.001$), and a significant week × day interaction (Wald $\chi^2(7)$=8.75 × $10^{13}$, $p < 0.001$), indicating that the distribution of LIT varied markedly across weeks and days of the microcycle. EMMs showed substantial week-to-week fluctuations in LIT, with higher values observed in initial (e.g. 2 and 3) mid-season weeks (e.g., 8) and lower values toward the end of the season (e.g., week 12). Within the microcycle, LIT was consistently higher on post-match days (MD+2 and MD+3) and lower on MD (Table S2).

The LMM fully confirmed the GEE results, showing a significant effect of week ($F(11,426)$= 81.51, $p < 0.001$), a significant effect of training day ($F(5,426)$= 1727.18, $p < 0.001$), and a significant week × day interaction ($F(55,426)$= 85.79, $p < 0.001$). The model demonstrated an excellent explanatory capacity, with a marginal $R^2$ of 0.962 and a conditional $R^2$ of 0.966, indicating that fixed effects explained the vast majority of the variance in LIT.

Moderate-intensity training (MIT)

The GEE analysis revealed a significant main effect of in-season week on MIT (Wald $\chi^2(8)$ = 3.6× $10^{11}$, $p < 0.001$), a significant main effect of training day (Wald $\chi^2(5)$ = 1032.24, $p < 0.001$), together with a significant week × training day interaction (Wald $\chi^2(8)$ = 4.06 × $10^{12}$, $p < 0.001$), demonstrating that the distribution of MIT within the microcycle varied substantially across in-season weeks. EMMs indicated higher MIT on post-match days (MD+2 and MD+3) and lower values on MD−1 (Table S2), although the magnitude of these differences was not consistent across weeks, as reflected by the significant interaction.

The LMM fully confirmed the GEE findings, revealing a significant effect of week ($F_{(11,426)} = 3.31$, $p < 0.001$), a significant effect of training day ($F_{(5,426)} = 171.55$, $p < 0.001$), and a significant week × day interaction ($F_{(55,426)} = 10.72$, $p < 0.001$). The model showed a high explanatory capacity, with a marginal $R^2$ of 0.719 and a conditional $R^2$ of 0.756.

High-intensity training (HIT)

The GEE analysis revealed a significant main effect of in-season week on HIT (Wald $\chi^2(8) = 5.66 \times 10^{12}$, $p < 0.001$), a significant main effect of training day (Wald $\chi^2(5) = 466.36$, $p < 0.001$), together with a significant week × training day interaction (Wald $\chi^2(7) = 4.63 \times 10^{13}$, $p < 0.001$), demonstrating that the distribution of HIT within the microcycle differed substantially across in-season weeks. EMMs showed that HIT was predominantly accumulated on MDs and early post-match sessions (MD+2), with consistently lower values observed on the day preceding competition (MD−1) (Table S2). However, the magnitude of HIT accumulation varied considerably across weeks, as highlighted by the significant interaction effect, with selected weeks displaying marked peaks in MD's HIT and others characterised by a more attenuated weekly HIT accumulation.

LMM confirmed the GEE results, showing a significant effect of week ($F_{(11,426)} = 6.11$, $p < 0.001$), a significant effect of training day ($F(5,426)= 227.55$, $p < 0.001$), and a significant week × day interaction ($F(55,426) = 4.87$, $p < 0.001$). The model demonstrated a high explanatory capacity, with a marginal $R^2$ of 0.730 and a conditional $R^2$ of 0.751.Table S2. Pairwise comparisons of regular in-season within-microcycle training-day internal load variables in outfield players

| Position | Variable | Training days | Pairwise comparison |
|---|---|---|---|
| **Outfielders** | | | |
| | **sRPE (AU)** | | |
| | | **MD+2** | MD+3 (< .001), MD-3 (< .001), and MD-1 (< .001) |
| | | **MD+3** | vs all (< .001) |
| | | **MD-3** | vs all (< .001) |
| | | **MD-2** | MD+3 (< .001), MD-3 (< .001), and MD-1 (< .001) |
| | | **MD-1** | vs all (< .001) |
| | | **MD** | MD+3 (< .001), MD-3 (< .001), and MD-1 (< .001) |
| | **LIT (min)** | | |
| | | **MD+2** | vs all (< .001) |
| | | **MD+3** | vs all (< .001) |
| | | **MD-3** | vs all (< .001) |
| | | **MD-2** | vs all (< .001) |
| | | **MD-1** | vs all (< .001) |
| | | **MD** | vs all (< .001) |
| | **MIT (min)** | | |
| | | **MD+2** | MD+3 (< .001), MD-2 (< .001), MD-1 (< .001), and MD (< .001) |
| | | **MD+3** | MD+2 (< .001), MD-3 (< .001), MD-1 (< .001), and MD (.045) |
| | | **MD-3** | MD+3 (< .001), MD-2 (< .001), MD-1 (< .001), and MD (< .001) |
| | | **MD-2** | MD+2 (< .001), MD-3 (< .001), MD-1 (< .001), and MD (.003) |
| | | **MD-1** | vs all (< .001) |

| | | |
|---|---|---|
| | **MD** | MD+3 (.045), MD+2 (< .001), MD-3 (< .001), MD-2 (.003), and MD-1 (< .001) |
| **HIT (min)** | | |
| | **MD+2** | vs all (< .001) |
| | **MD+3** | MD+2 (< .001), MD-3 (< .001), MD-1 (< .001), and MD (< .001) |
| | **MD-3** | vs all (< .001) |
| | **MD-2** | MD+2 (< .001), MD-3 (< .001), MD-1 (< .001), and MD (< .001) |
| | **MD-1** | vs all (< .001) |
| | **MD** | vs all (< .001) |

AU = arbitrary units; min = minutes; sRPE = session rating of perceived exertion; LIT = low-intensity training; MIT = medium-intensity training; HIT = high-intensity training; n.s. = not significant. MD±X = relative day from match day (MD).

*Congested In-season microcycles*

Session rating of perceived exertion (sRPE)

The GEE analysis revealed a significant main effect of congested week (Wald $\chi^2(4)$=110,448, p<0.001), a significant main effect of training day (Wald $\chi^2(4)$= 1060,67, p<0.001), and a significant week × day interaction (Wald $\chi^2(4)$= 5,8 x 1013, p < 0.001), indicating that perceived internal load varied substantially across the congested schedule and was unevenly distributed within the weekly microcycles. EMMs showed that sRPE

was generally highest on MDs and early post-match sessions, while markedly lower values were observed on the pre-MDs, although the magnitude of these differences differed across weeks (Table S3). Notably, Week 4 consistently exhibited the lowest sRPE values across most training days, whereas Weeks 1, 3, and 5 were characterised by higher perceived loads, particularly on MDs.

The LMM confirmed the GEE results, showing a significant effect of congested week ($F(4,144.03) = 8.94$, $p < 0.001$), a significant effect of training day ($F(4,144.0) = 41.22$, $p < 0.001$), and a significant week × day interaction ($F(16,144.03) = 5.61$, $p < 0.001$). The model demonstrated a moderate-to-high explanatory capacity, with a marginal $R^2$ of 0.588 and a conditional $R^2$ of 0.645, indicating that fixed effects accounted for the majority of the variance in sRPE.

*Low-intensity training (LIT)*

The GEE analysis revealed a significant main effect of congested week (Wald $\chi^2(4) = 782.41$, $p < 0.001$), a significant main effect of training day (Wald $\chi^2(4) = 4757.99$, $p < 0.001$), and a significant interaction (Wald $\chi^2(7) = 1.02 \times 10^{14}$, $p < 0.001$), indicating marked week-to-week variations in LIT distribution and substantial within-week modulation during fixture congestion. EMMs showed that LIT was highest during congested Week 5 (70.9 ± 1.5 min), which was significantly greater than all other weeks (all $p < 0.001$), whereas Weeks 1 and 4 exhibited the lowest overall LIT exposure. Across training days, LIT values were highest on MD+2/−1 and MD+2/−2, while MDs were characterised by substantially lower LIT accumulation (all $p < 0.001$) (Table S3). The significant interaction reflected week-specific redistribution patterns, with Week 5 showing particularly elevated LIT values on recovery-oriented days, whereas earlier congested weeks displayed a more compressed within-week distribution.

The LMM confirmed the GEE results, revealing a significant effect of congested week ($F(4,144.02) = 48.66$, $p < 0.001$), a significant effect of training day ($F(4,143.99) = 171.91$, $p < 0.001$), and a significant week × day interaction ($F(16,144.02) = 21.41$, $p < 0.001$). The model demonstrated a high explanatory capacity, with a marginal $R^2$ of 0.853 and a conditional $R^2$ of 0.879.

Moderate-intensity training (MIT)

GEE analysis revealed significant main effects across congested weeks (Wald $\chi^2(4) = 24.93$, $p < 0.001$) and within-microcycle training days (Wald $\chi^2(4) = 341.29$, $p < 0.001$), and their interaction (Wald $\chi^2(8) = 1.50 \times 10^{14}$, $p < 0.001$), indicating that the daily distribution of MIT differed substantially across congested weeks. EMMs showed that MIT were generally higher in the mid congested weeks, with week 3 displaying the highest mean MIT (EMM = 15.14 ± 1.54), while lower values were observed in weeks 4 and 5. Regarding within-microcycle days, the highest MIT accumulation occurred on MD+2/−1 and MDs, whereas MD−1 and MD+2/−2 were associated with substantially lower MIT time (Table S3).

LMM confirmed the GRR results. Significant fixed effects were observed for congested weeks ($F(4, 144.01) = 3.26$, $p = 0.014$), within-microcycle days ($F(4, 143.99) = 15.15$, $p < 0.001$), and their interaction ($F(16, 144.01) = 3.86$, $p < 0.001$). The model explained a meaningful proportion of variance, with a marginal $R^2$ of 0.39 and a conditional $R^2$ of 0.50.

High-intensity training (HIT)

GEE revealed a significant main effect of congested weeks (Wald $\chi^2(4) = 21.293$, $p < 0.001$), a significant main effect of within-microcycle training days (Wald $\chi^2(4) = 215.723$, $p < 0.001$), and a highly significant week × day interaction (Wald $\chi^2(7) = 2{,}3 \times 1010$, $p < 0.001$). EMMs indicated that HIT varied across congested weeks, with the highest values observed during week 3 (EMM = 10.43 ± 0.91 min) and progressively lower values in weeks 4 and 5 (week 4: 9.23 ± 0.81 min; week 5: 8.46 ± 0.86 min). Pairwise comparisons showed that week 3 differed significantly from weeks 5 ($p = 0.025$) and 4 ($p = 0.004$), while other contrasts were generally not

significant after adjustment. A pronounced effect of within-microcycle training days was observed, with markedly higher HIT accumulation on both MDs (EMMs ≈ 16.7–17.1) compared with other training days (EMMs ≈ 3.6–6.5), with most pairwise comparisons reaching statistical significance ($p<0.001$) (Table S3). The significant interaction reflected substantial modulation of daily HIT across congested weeks.

The LMM partially confirmed these findings. The LMM showed a non-significant main effect of congested weeks ($F(4, 144.02)= 1.282$, $p = 0.280$), a strong main effect of within-microcycle days ($F(4, 144.00)= 105.885$, $p < 0.001$), and a significant congested week × day interaction ($F(16, 144.02) = 2.850$, $p < 0.001$). The model demonstrated good explanatory power, with a marginal $R^2$ of 0.685 and a conditional $R^2$ of 0.748.

Table S3. Pairwise comparisons of congested in-season within-microcycle training-day internal load variables in outfield players

| Position | Variable | Training days | Pairwise comparison |
|---|---|---|---|
| **Outfielders** | | | |
| | **sRPE (AU)** | | |
| | | **MD+2/-2** | MD-1 (< .001), MD1 (< .001), and MD2 (.033) |
| | | **MD-1** | vs all (< .001) |
| | | **MD1** | MD+2/-2 (< .001), MD-1 (< .001), and MD+2/-1 (< .001) |
| | | **MD+2/-1** | MD-1 (.004), MD1 (< .001), and MD2 (.001) |
| | | **MD2** | MD+2/-1 (.001) and MD-1 (< .001) |
| | **LIT (min)** | | |
| | | **MD+2/-2** | vs all (< .001) |
| | | **MD-1** | vs all (< .001) |
| | | **MD1** | vs all (< .001) |
| | | **MD+2/-1** | vs all (< .001) |
| | | **MD2** | vs all (< .001) |
| | **MIT (min)** | | |
| | | **MD+2/-2** | MD-1 (< .001) and MD+2/-1 (.002) |
| | | **MD-1** | vs all (< .001) |
| | | **MD1** | MD-1 (< .001) and MD+2/-1 (<.001) |
| | | **MD+2/-1** | MD-1 (.006) and MD1 (<.001) |
| | | **MD2** | MD-1 (< .001) |
| | **HIT (min)** | | |
| | | **MD+2/-2** | MD-1 (.001), MD1 (< .001), MD+2/-1 (.002), and MD2 (< .001) |
| | | **MD-1** | MD+2/-2 (<.001), MD1 (< .001), and MD2 (< .001) |
| | | **MD1** | MD+2/-2 (< .001), MD-1 (< .001), and MD+2/-1 (< .001) |
| | | **MD+2/-1** | MD+2/-2 (.002), MD1 (< .001), and MD2 (< .001) |
| | | **MD2** | MD+2/-2 (< .001), MD-1 (< .001), and MD+2/-1 (< .001) |

AU = arbitrary units; min = minutes; sRPE = session rating of perceived exertion; LIT = low-intensity training; MIT = medium-intensity training; HIT = high-intensity training; n.s. = not significant. MD±X = relative day from match days (MDs).
